# No pandemic preparedness and research without gender equality

**DOI:** 10.1136/bmj.p1213

**Published:** 2023-06-07

**Authors:** Jocalyn Clark, Asha George, Rajat Khosla

**Affiliations:** 1 The BMJ, London, UK; 2 School of Public Health, University of the Western Cape, Cape Town, South Africa; 3 International Institute for Global health, United Nations University, Kuala Lumpur, Malaysia

The covid-19 emergency may have been declared over, but its effects are not. Pre-existing inequities worsened during the pandemic, and the crisis has hardened societal fault lines. Sex and gender mark many of these. Early on, sex and gender featured visibly as men seemed at higher risk of infection and hospital admission, and women of longer term illness and caregiving burdens. Over time, it became evident that covid-19 was exacerbating multiple and intersecting vulnerabilities, with substantial effects on women and girls: increased care burdens, amplified gender based violence during lockdowns, catastrophic drops in income and employment for women and families, disrupted essential health services, and school closures that heightened risk of unintended pregnancies and permanent dropouts.

By early 2023 it was clear covid-19 had wiped out hard won gains on gender equality. And while we are still living with the effects of covid-19, worsening planetary threats—including war, the climate emergency, and economic uncertainties—are creating profound global instability. Sex and gender must define our responses to these and future crises.

To further that aim, a new BMJ collection of articles (www.bmj.com/gender-and-pandemic-response) lays out a shared research agenda for sex, gender, and health for covid-19 and future pandemics and crises. A partnership of *The BMJ,* the International Institute for Global Health at United Nations University, and the School of Public Health at the University of Western Cape, and supported by the Bill and Melinda Gates Foundation, the collection is the product of a large, multinational research initiative. Its goal has been to strengthen covid-19 responses as well as to ensure future pandemic response and research are more effective in making sustained advances in gender equality around the world.

## Maintaining visibility

An upside of covid-19 is that it led global health leaders and policy makers to give greater importance to sex and gender. In the era of “polycrises,” this attention is now at risk. The collection aims to strengthen the recognition of sex and gender as integral to all aspects of global health. We need to counter impulses to “return to normal” after covid-19. Retreating from progressiveness in responding to new or persistent global threats would be damaging not only to women and girls, but to the visibility of feminist engagement in the health sector and the ability to recover progress towards the sustainable development goals.

The research initiative informing the collection has remarkable scope and geography: over 1000 participants, a mix of established experts and emerging leaders, largely from low and middle income countries, and about 75% women. This effort yielded a shared research agenda on sex differences and gender equality priorities linked to diverse perspectives and experience.^1^ It emphasises the importance of addressing the basics in gender and health, such as sex disaggregated data and sex specific needs, while also advancing broader goals to establish gender justice within health and social policy and programmes**.**

A second feature is the intersectional feminist methodology used, which consisted of crowdsourcing a collaborative engagement process anchored in the global south. In addition to standard prioritisation surveys, feminist principles mindful of intersectional power dynamics underpinned how research gaps were reviewed, research questions were framed, publications were developed, and engagements were undertaken, emphasising distributed and consultative leadership. Established gender and health advocates collaborated with those working in fields where feminist analysis is just emerging, such as researchers and advocates from the basic sciences. Vijayasingham and colleagues lay out the resulting framework for advancing sex and gender analyses in scientific research, clinical trials, and patient rights, arguing that industry, innovators, and investors can benefit from including sex and gender related factors in all product development strategies.^2^

The collection highlights how regional strategies are crucial to advancing gender and health. In a deep exploration of the African response to covid-19, for example, Bello and colleagues argue that while the continent’s efforts to respond to women’s immediate needs during the pandemic have been laudable, resilience to future crises requires investment in sustainable strategies to advance gender equality, especially in relation to gender based violence, social protection, and community and civil society mobilisation.^3^

Recognising that marginalisation is not uniformly experienced during crises, and changes over time, responses to such events need to be inclusive. As Mothupi and colleagues describe, the voices of people most central to co-creating health, including healthcare users and health workers, must be meaningfully integrated in health system transformations to prepare for the next pandemic and future crises.^4^

The collection illuminates the importance of sex and gender to the experience of crises like covid-19, as well as the quality and effectiveness of the available responses. Centring the principles of collectivity, inclusivity, and intersectionality, we hope the articles contribute to other collaborative efforts for developing sex and gender responsive research and preparedness for future pandemics and crises.^5^ ^6^ ^7^ Most importantly, we hope the work establishes a model for co-produced feminist and decolonial approaches to global health collaboration, including the use of digital platforms for engagement, inclusive conversation, and open consensus building. More inclusive ways of working are critical for global health as it faces further uncertainties in the aftermath of covid-19.

## Notes

Provenance and peer review: Commissioned; not externally peer reviewed.
